# Willingness to pay for municipality hospital services in rural Japan: a contingent valuation study

**DOI:** 10.1186/1756-0500-4-177

**Published:** 2011-06-07

**Authors:** Takayoshi Terashita, Hiroshi Muto, Toshihito Nakamura, Katsuhiko Ogasawara, Masaji Maezawa

**Affiliations:** 1 Department of Healthcare Systems Research, Graduate School of Medicine, Hokkaido University, Sapporo, Japan; 2 Faculty of Health Sciences, Graduate School of Health Sciences, Hokkaido University, Sapporo, Japan; 3 Department of Medical Informatics, Hokkaido University Hospital, Sapporo, Japan

## Abstract

**Background:**

The Japanese healthcare system has undergone reforms to address the struggles that municipality hospitals face. Reform guidelines clearly define criteria for administrative improvement. However, criteria to evaluate the demand for healthcare provisions in rural Japan, including the needs of rural residents for municipality hospitals in particular have not been specified. The purpose of this paper is to measure residents' willingness to pay (WTP) for municipality hospital services using the contingent valuation method, and to evaluate municipality hospital valuation on the basis of WTP. K town, located in the Hokkaido prefecture of Japan, was selected as the location for this study. Participants were recruited by a town hall healthcare administrator, hospital and clinic staff, and a local dentist. Participants were asked what amount they would be willing to pay as taxes to continue accessing the services of the municipality hospital for one year by using open-ended questions in face-to-face interviews.

**Findings:**

Forty-eight residents were initially recruited, and 40 participants were selected for the study (response rate 83%). As compared to K town's population, this data slanted toward the elderly, although there was no significant difference in frequency among the characteristics. The median WTP was estimated at 39,484 yen ($438.71), with a 95% confidence interval 27,806-55,437 yen ($308.95-615.96). Logistic regression revealed no significant factors affecting WTP.

**Conclusions:**

If the total amount of residents' WTP for the municipality hospital were to be estimated by this result, it would calculate with 129,586,000 yen ($1,439,844). This is approximately equal to the amount of money to be transferred from the general account of the government of K town, more than one-half of the town tax of K town, and about two-fold in comparison to Japan as a whole. This showed that K town's residents placed a high valuation on the municipality hospital, which nearly equalled the amount that the K town government provided to the municipality hospital to cover its annual deficit. K town residents had come to expect not only general clinical practice, but also emergency medical services and night practice provided by their own town's municipality hospital. WTP can be used as a measure of hospital evaluation because it reflects the importance of the hospital to the residents in its region.

## Introduction

The Japanese healthcare system is very efficient and offers high quality care. According to the Organization for Economic Cooperation and Development (OECD) Health Data, in 2007, Japanese medical costs per Gross Domestic Product (GDP) were 8.1%, half that of the United States (16.0% of GDP). Despite the low medical cost per GDP, Japanese life expectancy is the highest in the world [1,2]. However, most municipality hospitals in Japan have struggled to maintain standards of healthcare in light of changes in the local healthcare system, such as fewer patients, a lack of doctors and healthcare workers, as well as a reduction in medical reimbursement [3]. According to a 2007 annual report by the local public sector, municipality hospitals' average losses were 75.1% (501 of 667 hospitals), and the total deficit reached 200 billion yen [4]. Japanese municipality hospitals play a valuable role in rural Japan by providing local people access to medical services [5]. The municipality hospital's main functions are to provide general medical services at rural locations where building a private hospital would be difficult, to provide medical treatment of deficit department and specialty departments, such as emergency care, pediatrics, perinatal, psychiatric, and disaster medicine, and to provide advanced medical technology. Currently, the burden to fund the municipality hospitals falls on the local governments.

In 2008, the Japanese Cabinet set forth a policy for public hospital reform as one of its countermeasures against the ailing economy [3]. The goal was to increase the efficiency of public hospitals by reducing the number of beds downsizing the hospital to clinic status, and closing down inefficient public hospitals. However, some public hospitals, despite inefficiencies, must be maintained to meet the obligations of providing healthcare in rural areas. Therefore, public hospital reform will need to be restructured after clarification of the roles of individual public hospitals. The reform guidelines outlined criteria for measuring administrative improvements as a means to evaluate management efficiency. The ratio of income to expenditure, the medical practice-profit ratio compared to staff salary costs, and the sickbed utilization rate were used to gauge efficiency. However, criteria to evaluate the basic demand, or need, for rural healthcare services are not specified. The need for rural healthcare services is related to the value rural residents place on access to a municipality hospital, and can be evaluated by measuring that value [6].

## Background

There is a market value and a non-market value intrinsic to hospital valuation. The market value of a hospital is determined by evaluating profit. The non-market value is more difficult to evaluate. If the local residents feel that the hospital management is inefficient or that the residents have little use for the hospital's services, it would have a low valuation. On the other hand, if the hospital offered emergency care or night services or if the residents used its services often, it would have a high valuation. Those valuations, such as expectancy and complacency derived from hospital existing in local area for residents, are the non-market value. The contingent valuation (CV) method is used to evaluate this non-market value, and can evaluate by using comparable measurements such as monetary value [7]. In this approach, the investigators provided participants with a fact sheet and a hypothetical situation with research questions. The participants were asked their willingness to pay (WTP) on the basis of the hypothetical situation. This approach has been used in environmental research, policy assessments, and other studies. Many CV studies have been applied to the healthcare realm. Klose reviewed studies published before 1998 and showed the validity of CV studies as well as some sources of bias applied to healthcare [8]. Ogasawara expounded health economics in generally, and described the application of CV to healthcare in Japan [9,10]. Previous studies have addressed various topics, such as treatment of diseases, health insurance, and medical services. For example Frew et al. investigated the general practitioners' WTP for colorectal cancer screening in the UK [11]. They found that the WTP was similar to the resource costs for executing the screening program. The WTP is a relative value, and the evaluation is carried out by comparison with other programs of the same quality, or comparison to programs that present an alternative. This suggests that the valuation can be determined by comparing costs. Haefeli et al. investigated the WTP for spinal surgery in the UK using a bootstrapping method helpful in estimating a 95% confidence interval for a small sample size [12]. Ringburg et al. in their evaluation of the WTP for helicopter emergency medical services in the Netherlands evaluated the WTP not by a CV method but by a discrete choice experiment [13]. This method is able to ask the value of multiple programs for the same group of participants. However, this method could not be applied to our study because we targeted only the municipality hospital.

For study of the health policies in Asian countries other than Japan, Asgary et al., Barnighausen et al., Dror et al., Lang et al., and Lofgren et al. investigated the WTP for health insurance in Iran, China, India, Taiwan, and Vietnam, respectively [14-18]. Asgary et al. investigated the benefit assuming WTP as the need for healthcare services [14]. Lang et al. concluded that the result of the WTP is helpful in decision making on health policies because the CV method is similar to that of a referendum [15]. It is very significant for health policy in that it respects the will of residents. Barnighausen et al. compared WTP by difference in occupation and showed that it is affected by the association of occupation with the income it provides [16]. Dror et al. found that participants' income, education, and sex affected their WTP [17]. Hence, income, education, sex, and employment are all cited as the factors affecting WTP. Lofgren et al. used an interview to obtain responses [18]. When the interview method is used, the participants are able to understand the contents of the questionnaire clearly, and their responses become high quality data.

Our study uses examples specific to Japan. Yasunaga, the foremost Japanese expert on health economics, investigated the WTP using the CV method for several scenarios. His research team addressed the WTP for a cancer screening program using positron emission tomography, medical services for cardiovascular disease, as well as healthcare services for common cold, retinal detachment, and myocardial infarction in Japan [19-21]. Other authors have investigated the WTP for different features of the health care system: Hario for the home health support system [22], Ogasawara for the pediatric telemedicine system [23], and Takemura for the gastric cancer screening program in community health services [24]. The WTP values of previous studies in Japan serve as useful references for comparison with our study of the WTP, because they used the same framework of medical administration system. Additionally, in any CV study conducted in Japan, the researcher should choose the method of payment carefully because the contribution does not have roots in our culture. However, there is presently no study that addresses residents' WTP for municipality hospital to our knowledge, although we picked up the references widely.

The purpose of this paper is to measure residents' WTP for municipality hospital by using the CV method and to evaluate a municipality hospital on the basis of that WTP.

## Methods

### Study location and hospital

We selected K town in the Hokkaido prefecture of Japan as the location for this study because of its decreasing population, higher age-adjusted medical cost rate, and because it has a faltering municipality hospital.

The population of K town is 3,282 (1,583 households), and is 47.0% male. Senior residents 60-69 years of age comprise 12.9% of population, and 24.8% are over 70 years of age. K town's industry is a dairy husbandry, farming, and forestry. The town's medical facilities consist of one municipality hospital and four small clinics, which provide services in the specialties of general surgery, orthopedics, pediatrics, and internal medicine. Three middle-size hospitals are located within 50 kilometres of the town. These hospitals tend to patients who could not secure care in K town or who need emergency care.

The K town municipality hospital has 40 sickbeds and a department for general surgery, orthopedics, and internal medicine. The hospital's staffs consist of 2 doctors, 12 nurses, and 4 other healthcare workers. The sickbed utilization rate of K town's municipality hospital is 27.7%, one-third the Japanese national average of 82.2%. The hospital's rate of employment cost is 76%. This rate is higher than the Japanese national average of 55%. While the annual cost to provide medical services is approximately 47 million yen, the K town hospital's income is only approximately 30 million yen, leaving an annual deficit of 17 million yen. The K town government has historically covered this deficit by providing approximately 20 million yen to the hospital annually. Although in 2007 the K town's municipality hospital was not operating at a loss, the hospital is vulnerable to losses. A reduction in the number of patients would result in a decrease in the hospital's income, and it was at risk of becoming unsustainable. To approach this issue, the cooperation of K town's government was requested, in particular for the introduction of participants and the contribution of incentive for them. K town's government representatives agreed to assist in the study.

### Questionnaire

To elicit the value the town's residents place on municipality hospital, each participant was presented with an objective fact sheet and a hypothetical situation. The fact sheet included the following: the municipality hospital's mission, information on the Japanese government's restructuring program for the hospital, and the financial condition of the municipality hospital. In the hypothetical situation, participants were asked to imagine a situation without the municipality hospital. Participants were then presented with a hypothetical description of the medical care provided under that scenario. Finally, the participants were asked how much they were willing to pay in additional taxes to maintain the municipality hospital for one year. To account for external factors that might impact WTP in this study, participants were asked to provide their age, sex, educational background, employment status, family structure, affiliated health insurance, and level of medical services use per month. However, participant's income was not collected in this study because K town's government would not grant permission to do so.

Data collection was conducted by open-ended questions in a face-to-face interview. Generally, CV study employed a double-bounded dichotomous choice contingent valuation approach using a paper-based questionnaire as the data collection method. However, such questionnaires often provide a low collect rate, which becomes an issue when the object is to get a high response rate in small population. A poorly explained questionnaire may lead to inappropriate responses because respondents may misinterpret or misunderstand the questions. In addition, the double-bounded dichotomous choice contingent valuation approach cannot elicit individual WTPs for the residents. This brought up the consideration whether zero WTPs were to be included in analysis data when responses were zero WTP. Conversely, none of those disadvantage are inherent in open-ended questions using a face-to-face interview. Oscarson et al. conducted a CV study of caries preventive care using 52 samples [25]. Despite the small sample size, they concluded that their method was appropriate for investigation and analysis. Because it elicited the individual WTPs by using open-ended questions, and estimated a certain value. Open-ended questions can elicit an individual WTP, and the face-to-face interview can provide each participant with a thorough explanation. Thus, we determined that open-ended questions using a face-to-face interview were an appropriate approach in our setting, although the sample size was small. The questionnaire we used is provided in the Appendix.

### Participants

Participants were recruited by a town hall healthcare administrator, staff members of the hospital and clinics, and a dentist in K town. The rehabilitation room at the municipality hospital and at one of the clinics in town, a participant's working office, or a participant's home were used for the interviews. As an incentive, participants were offered a coupon for a thermal bath worth 500 yen. The study was performed between September 3 and 24, 2009. Forty-eight residents were initially recruited, and 40 participants agreed to participate in the study (response rate 83%).

As ethical considerations, all participants provided informed consent before starting the interview, and participants who declined to respond were not included in the study. This study was approved by the institutional review board of the Graduate School of Medicine, Hokkaido University, Japan.

### Data analysis

The overall characteristics of participants are shown in Table 1. We organized the participants into groups depending on their WTP, and show the characteristics of each group (Table 2). The residents' WTP for the municipality hospital was evaluated from individual WTPs using a logit model, based on the random utility model. The log-linear function was used as the function type. The formula used in this study was:

**Table 1:** Participants' characteristics (n = 40)

	n
Sex	
Male	21
Female	19

Age	
< 59 years of age	12
60-69 years of age	12
> 70 years of age	16

Educational background	
Compulsory education	13
High school	19
Professional education (including university and professional school)	8

Family structure	
Single	5
2 persons	24
> 3 persons	11

Employment	
Yes	19
No	21

Health insurance	
National Health Insurance	14
Social Insurance	14
Old-Old National Health Insurance	12

Access to medical service	
> once monthly	16
Several times	24

Place of interview	
Town's hospital	13
Clinic	7
Participants' office	7
Other	13

Combination measure for income	
low	12
middle	20
high	8

Individual WTP	
Zero	4
< 50,000 yen	18
50,000-100,000 yen	13
> 100,000 yen	5

**Table 2:** Comparison between WTP group and each demographic factor

		WTP group (yen)				
		< 20,000	20,000-33,000	33,000-60,000	> 60,000	Total
Sex	Male	5	4	9	3	21
	
	Female	8	3	5	3	19

Age	< 59 years of age	7	0	3	2	12
	
	60-69 years of age	0	2	8	2	12
	
	> 70 years of age	6	5	3	2	16

Educational status	Compulsory education	4	5	3	1	13
	
	High school	5	2	8	4	19
	
	Professional education	4	0	3	1	8

Family structure	Single	0	2	3	0	5
	
	2 persons	8	5	8	3	24
	
	> 3 persons	5	0	3	3	11

Employment	Yes	6	3	7	3	19
	
	No	7	4	7	3	21

Medical insurance	Social Insurance	5	1	6	2	14
	
	National Health Insurance	4	2	6	2	14
	
	Old-Old National Health Insurance	4	4	2	2	12

Access to medical institution	> once monthly	5	2	7	2	16
	
	Several times	8	5	7	4	24

Place of interview	Town's hospital	4	3	5	2	14
	
	Clinic	3	4	2	0	9
	
	Participants' office	4	0	3	0	7
	
	Other	2	0	4	4	10

Combination measure for income	Low income	3	3	4	1	11
	
	Medium income	6	4	7	4	21
	
	High income	4	0	3	1	8

Total		13	7	14	6	40

where Pr[yes] is the assenting rate i.e., the probability of saying "yes" to the WTP; WTPi is an individual WTP; a and b are coefficients. As the representative value for WTP, we assigned the median value. The median WTP, which means that one half of the participants approved a price, is used when considering fairness, and is calculated as -a/b. We estimated 95% confidence intervals (CI) using a bootstrapping method. This method is useful in studies with a small sample size [26]. One thousand loops of operation were repeated for samples. To identify factors affecting WTP, we used a logistic regression analysis, which is used when the dependent variable is from zero to one, such as a probability. However, we could not elicit the participant's incomes, which were shown in previous studies to be the most important factor affecting WTP [7,27]. Therefore, we substituted a combination measure for a participant's income by combining educational background, employment status and family structure. This is based on the assumption that higher education level, having employment and many family members indicate a higher income. We tested the relationship between WTP and the following variables: age, sex, educational background, employment status, family information, affiliated health insurance, the use of medical services per month, place for interview, and the combination measure for income. The statistical software R version 2.9.0 (The R Project for Statistical Computing, http://www.r-project.org/), was used for every analysis. Statistical significance was accepted at p < 0.05. The exchange rate was assumed to be 90 yen for one US dollar.

## Results

**Figure 1:**
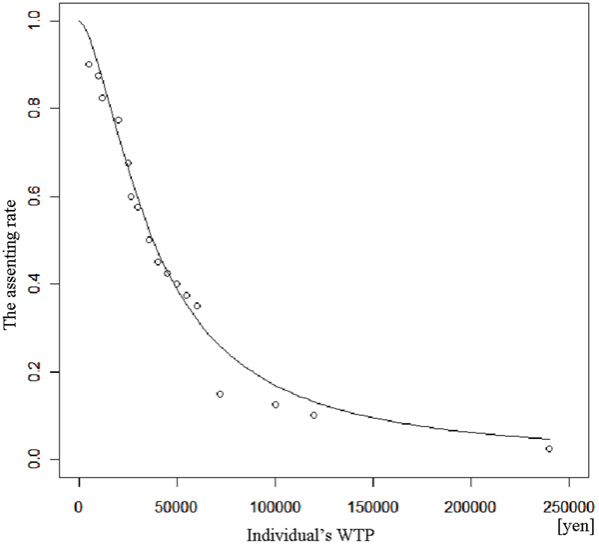
**Plot of individual WTP and estimated demand curve**. The median WTP was estimated to 39,484 yen ($438.71), with a 95% confidence interval of 27,806-55,437 yen ($308.95-615.96). The Nagelkerke's coefficient of determination was 0.972.

The overall participant characteristics are presented in Table 1. Maximum value of the individual WTP, based on participant responses, was 240,000 yen ($2,666.66), and minimum value was 0 yen. The mode value was 60,000 yen ($666.66). As compared to K town's population, this data slanted toward the elderly, although the sex ratio was at the same level. There was no significant difference in frequency among the characteristics. The comparisons among individual WTP groups divided by quartiles are shown in Table 2. There was a significant difference in the relationship between WTP groups and age using the Kruskal-Wallis test. In terms of the other factors, there were no significant differences. However, the results of the logistic regression analysis could not account for some significant factors. The estimated demand curve is presented in Figure 1. The median WTP was estimated as 39,484 yen ($438.71), with a 95% CI 27,806-55,437 yen ($308.95-615.96). The Nagelkerke's coefficient of determination was 0.972, and the fitting of the function was good. The results of the logistic regression analysis, shown in Table 3, do not account for some significant factors affecting this study.

**Table 3:** Results of logistic regression analysis

		Estimate	Standard Error	p-value	
Intercept		17.7	1.21	p < 0.01	*
WTP		-1.63	0.10	p < 0.01	*

Sex	Male	-0.0944	0.12	0.45	

Age	> 70 years of age	-0.216	0.26	0.42	
	< 59 years of age	-0.0960	0.25	0.70	

Educational status	High school	-0.141	0.16	0.38	
	Professional education	-0.175	0.21	0.41	

Family structure	Single	-0.159	0.26	0.54	
	2 persons	-0.110	0.19	0.57	

Employment	Person with a job	-0.0178	0.14	0.90	

Medical insurance	National Health Insurance	-0.0424	0.22	0.85	
	Social Insurance	-0.117	0.35	0.73	

Access to medical institution	> once monthly	-0.0221	0.20	0.91	

Place of interview	Town's hospital	-0.0892	0.18	0.62	
	Participants' office	-0.0194	0.25	0.93	
	Other	-0.0955	0.17	0.59	

Combination measure for income	Low	-0.0841	0.26	0.75	
	Medium	-0.0204	0.23	0.93	

## Discussion

It could be concluded that K town's residents place a high valuation on the municipality hospital, in that the median hospital's WTP was estimated to be about 40,000 yen. First, we compared this value with other studies in Japan to evaluate the magnitude: the healthcare services for common cold at 3,140 yen/year [21]; the pediatric telemedicine system at 3,472 yen/year [23]; the cancer screening program with positron emission tomography at 7,480 yen/use [19]; the gastric cancer screening program in community health services at 8,571 yen/year [24]; the home health support system at 16,092 yen/year [22]; the healthcare services for cardiovascular disease at 7,878 yen/month [20]; and the healthcare services for retinal detachment and myocardial infarction at 234,465 yen/year and 942,480 yen/year [21], although it was difficult to draw a comparison given the difference in settings. The WTP in this study is higher than that for home health support systems and lower than that for healthcare services for cardiovascular disease in Japan. Viewed in this light, the value is high for conditions that have the potential to cause death or disability. K town's valuation is rather high event though it does not influence participants' life directly, because its municipality hospital has both emergency care and hospitalization services. In this regard, it must be noted that the sample slants toward the elderly. Generally, the elderly will have a degree of dependence on healthcare that is higher than that of other generations, which may have an important influence on the valuation. However, neither factor affected WTP. In addition, other important factors might not have been included in the variable set. For example, respondents' income should have been included as an affecting factor of WTP [7,27]. Since we did not obtain permission from the government to obtain participants' income for this study, we substituted the combination measure described previously for resident's income. This combination measure also had no effect on WTP. We thought that there was not the difference among responses, because most of K town's industry was a low income.

On the other hand, if the total amount of residents' WTP for K town's municipality hospital was estimated using this result, it is calculated with 129,586,000 yen ($1,439,844) (3,282 residents multiplied by 39,484 yen). According to the municipality hospital's income and expenditure report, the government of K town transferred 124,141,000 yen ($1,379,344) to the hospital to maintain services [3]. This value was almost equal to the calculated value. Furthermore, according to the account settlement of K town at the time, the town tax was 268,146,000 yen ($2,979,400). The calculated WTP could require more than half the town tax. In addition, according to the income statement for the municipality hospital in Japan, the mean of absolute profit without the provision from each local government was minus 65,992,000 yen ($733,244) [3]. The transferred money for K town's municipality hospital is about two-fold in comparison to Japan as a whole. This is to say that K town's residents expected the continuation of their own town's hospital even if they paid half the town tax in spite of unprofitable hospital judging from Japan as a whole. This could be interpreted as indicating that K town's residents place a high value on their town's municipality hospital. In this regard, it must be noted that K town's total valuation for the municipality hospital may be overestimated. In addition, the 95% CI is wide, and the total valuation is calculated by multiplying the total population by the individual WTP. The lower value of the CI is about 27,000 yen, and the total WTP is about 91,259,000 yen. The calculation would be more reliable if the number of households were multiplied by the household WTP than by multiplying the total population by the individual WTP.

From this aspect, it could be assumed that K town's residents place a very high valuation on their town's municipality hospital: they have come to expect the emergency medical service and night practice along with the general clinical services it provides. As mentioned previously, inefficient hospitals have been required to downsize or close as specified by the criteria in the public hospital reform guidelines for Japan. However, K town's hospital is absolutely necessary for the residents, and the reduction of the hospital function will place a burden on patients. Some criteria that can judge the need including the residents' valuation of the hospital are necessary. In other studies, patient satisfaction was used to measure the quality of healthcare and to evaluate hospitals. This measure was then used to determine the level of funding allocated to the hospital [28-30]. The residents' WTP for the municipality hospital in this study may be used to estimate the value of the hospital. In this regard, although we showed only the K town residents' high valuation in this study, it will be necessary to evaluate most hospitals, or at least all the hospitals associated with them local areas. Nonetheless, improvement will be required in the management of the K town's municipality hospital in light of possible future changes in the healthcare environment, such as a decline in the hospital's income resulting from a decreasing population, medical reimbursement fees, and tax allocation from the national government.

## Limitations of the present study

Four biases were considered limitations in this study [27]. First, a sampling bias may exist because of the low sample size and because some participants were recruited by healthcare professionals. As a result, the sample in this study might not be representative of K town's residents, and the elicited responses may have an inherent bias. To obtain an accuracy of 95% ± 5% while estimating the WTP for K town's municipality hospital, 343 participants would be needed. However, with the face-to-face interview method, large sample sizes are difficult to obtain. In this study, we thought that the quality of responses was ensured by using face-to-face interviews, and that is supported by the low percentage of participants who refused to participate, at a response rate of 83%. However, it is necessary for the sample size to be large enough to conduct follow-up research using the questionnaire based on this study.

Second, a reporting bias may result from the face-to-face interview. The investigators introduced themselves as university researchers and presented the participants with a letter from the local government endorsing the study. Participants may have been eager to satisfy the investigators and responses may have been biased thereby.

Third, there may have been a lead-time bias, in which the responses provided by the participants were affected by when the questions were asked. The Japanese House of Representatives election was held one week prior to the first day of questioning. The result of election changed the Japanese government administration. Because CV method measures participants' subjective evaluation, there may have been a bias in the responses. The participants may answer a lower value if they have a bad impression in regard to previous health policy, or they may answer a higher value if they have good wishes for new government. However, since the health policy did not change substantially during one week, it was assumed that the affection on this study was little.

Last, the results may be affected a selection bias, because participants are enrolled after having already had their contact with the municipality hospital. Persons who are already exposed to a measuring object may give the different valuation compared to them who are not exposed. However, it is difficult to cut this bias in this study setting, because there are very few residents without receiving the municipality hospital's services once in K town. In the results of place for interview, the estimated WTP was not affected. Therefore, there was no the affection of this bias, or there was the strong influence overall.

## Appendix

### Questionnaire for contingent valuation research, including fact sheet and hypothetical situation

[Present status of the municipality hospital]

The municipality hospital plays an important role in preserving local healthcare as an essential medical institution in local areas. However, in recent times, most municipality hospitals in Japan have faced very difficult circumstances in which to continue their business of providing healthcare: business conditions have failed as a result of reduction in medical reimbursement fees, lack of doctors and nurses, and other considerations. According to reform guidelines, the national government decided that miniaturization of management for hospitals that have low statistical measures, such as the sickbed use rate, would be effective. K town's municipality hospital has 40 sickbeds and has departments of general surgery, orthopedics, and internal medicine. The staffs consist of 2 doctors, 12 nurses, and 4 other healthcare workers. The rate of sickbed utilization rate in K town's hospital is 27.7%, far lower than the national average (82.2%) in Japan. The rate of employment cost in K town's hospital is 76%, which is far higher than the national average (55%) in Japan. While costs of medical practice are about 47 million yen, the hospital's income obtained by medical practice is only about 30 million yen, and the annual deficit balance is about 17 million yen. K town's government has covered this deficit balance by about 20 million yen using money transferred from town's general accounts. Although K town's municipality hospital is not yet running at a loss, it may not continue to do remain viable if hospital income decreases by reason of reduction number of patients and other reasons as well.

[Hypothetical situation]

Please imagine the situation without a municipality hospital in the present K town.

General medical system shift:

For general outpatient service, you will go to K town's other clinics or to medical institutions in other towns. If you travel to medical institutions in other towns, you will use public transportation or taxicab. For hospital admission, you will go to a hospital in another town with sickbeds.

Particular medical system shift:

For mild emergencies, you will visit K town's clinics. For serious emergencies, you will visit a hospital in another town that has an emergency department. You will also go to a hospital in another town that has an emergency department because K town's clinics are not open during the night time. For consultation with special departments (for example, ophthalmology, urology, neurosurgery, and so on), you will go to hospitals or clinics in another town that has such a department.

[Question]

How much do you think that you would be willing to pay, if you had to pay money as tax increase to continue K town's municipality hospital?

A. yen.

## Competing interests

The authors declare that they have no competing interests.

## Authors' contributions

TT conceived the study, acquired the data, performed the analysis, interpreted the data, and wrote the first draft of the manuscript. HM helped to conceive the study, and provided technical support. TN and KO helped to interpret the data, and revised the manuscript critically. MM oversaw the study, and revised the manuscript critically. All authors read and approved the final manuscript.
